# 

**Published:** 1804-08-01

**Authors:** 


### To CORRESPONDENTS.

Communications are received from Dr. Trotter, Dr. Rodman, Mr. Edgar, Mr. Blake, and Candidus.

